# Real-Life Utilization of Criteria Guidelines for Diagnosis of Cardiac Sarcoidosis (CS)

**DOI:** 10.3390/jcm12165278

**Published:** 2023-08-14

**Authors:** Tal Gazitt, Fadi Kharouf, Joy Feld, Amir Haddad, Nizar Hijazi, Adi Kibari, Alexander Fuks, Edmond Sabo, Maya Mor, Hagit Peleg, Rabea Asleh, Devy Zisman

**Affiliations:** 1 Rheumatology Unit, Carmel Medical Center, Haifa 3436212, Israeldevyzisman@gmail.com (D.Z.); 2 Division of Rheumatology, University of Washington Medical Center, Seattle, WA 98195-6428, USA; 3 Rheumatology Unit, Hadassah Medical Center, Jerusalem 9112001, Israel; 4 Faculty of Medicine, Hadassah Medical Center, Jerusalem 9112001, Israel; rasleh@hadassah.org.il; 5 The Ruth and Bruce Rappaport Faculty of Medicine, Technion, Haifa 3200003, Israel; 6 Internal Medicine B, Carmel Medical Center, Haifa 3436212, Israel; 7 Department of Cardiology, Carmel Medical Center, Haifa 3436212, Israel; 8 Department of Pathology, Carmel Medical Center, Haifa 3436212, Israel; 9 Department of Radiology, Carmel Medical Center, Haifa 3436212, Israel; 10 Department of Cardiology, Hadassah Medical Center, Jerusalem 9112001, Israel

**Keywords:** cardiac sarcoidosis, 2014 HRS guidelines, disease presentation, cardiac biomarkers, CMR, PET-FDG

## Abstract

Despite the increasing recognition of cardiac involvement in systemic sarcoidosis, the diagnosis of cardiac sarcoidosis (CS) remains challenging. Our aim is to present a comprehensive, retrospective case series of CS patients, focusing on the current diagnostic guidelines and management of this life-threatening condition. In our case series, patient data were collected retrospectively, including hospital admission records and rheumatology and cardiology clinic visit notes, detailing demographic, clinical, laboratory, pathology, and imaging studies, as well as cardiac devices and prescribed medications. Cases were divided into definite and probable CS based on the 2014 Heart Rhythm Society guidelines as well as presumed CS based on imaging criteria and clinical findings. Overall, 19 CS patients were included, 17 of whom were diagnosed with probable or presumed CS based on cardiac magnetic resonance imaging (CMR) and/or cardiac positron emission tomography using ^18^F-Fluorodeoxyglucose (PET-FDG) without supporting endomyocardial biopsy (EMB). The majority of CS patients were male (53%), with a mean age of 52.9 ± 11.8, with CS being the initial manifestation of sarcoidosis in 63% of cases. Most patients presented with high-grade AVB (63%), followed by heart failure (42%) and ventricular tachyarrhythmia (VT) (26%). This case series highlights the significance of utilizing updated diagnostic criteria relying on CMR and PET-FDG given that cardiac involvement can be the initial manifestation of systemic sarcoidosis, requiring prompt diagnosis and treatment to prevent morbidity and mortality.

## 1. Key Messages

1. CS was the initial presentation of systemic sarcoidosis in the majority of CS cases in our case series.

2. Most cases of CS involved advanced AV conduction delays, with concurrent tachyarrhythmias in some.

3. Dual imaging using CMR and cardiac PET-FDG was instrumental in leading to CS diagnosis in cases where attempts at obtaining a histologic confirmation of sarcoidosis were unsuccessful, and also aided in physicians’ assessment of active myocardial inflammation.

4. The use of updated CS criteria is important for the prompt diagnosis and treatment of this condition.

## 2. Introduction

Sarcoidosis is an inflammatory granulomatous disease that can affect any body organ. Cardiac involvement is estimated to occur in approximately 20–25% of patients based on data from consecutive autopsies of patients with systemic sarcoidosis [1] and on right ventricular EMB histological data from systemic sarcoidosis patients [2], with only 5% of patients estimated to have clinically evident myocardial involvement [1,3,4]. Cardiac manifestations of sarcoidosis commonly include conduction defects, comprising septal involvement causing a high-grade atrioventricular block (AVB), re-entrant circuits involving areas of granuloma/fibrosis and culminating in tachyarrhythmias, especially ventricular tachycardia (VT), and extensive areas of biventricular involvement, clinically manifesting as heart failure with or without arrhythmias [5]. Cardiac involvement is associated with the highest morbidity and mortality in systemic sarcoidosis, requiring early diagnosis and therapy to mitigate disease progression and to prevent life-threatening events [6].

Notably, the diagnosis of CS remains a challenge as EMB has low sensitivity. For instance, one retrospective series of 851 patients with unexplained heart failure, covering the period from 2000 to 2009, showed an overall diagnostic yield of 25.5% for EMB, with 7/851 patients in this series having CS [7]. Another case series of 1235 patients with EMB performed for unexplained cardiomyopathy from 1982 to 1997 [8] showed a diagnostic yield of CS in only 25% (7/28) of patients who had a previous diagnosis of systemic sarcoidosis and three additional cases of CS in patients with other initial diagnoses. This low yield of EMB is thought to be due to the focal nature of this granulomatous disease [4], with additional concern regarding performing EMB due to the numerous possible cardiac complications associated with this procedure [9]. Given these diagnostic challenges, CS is occasionally diagnosed as a cause of cardiomyopathy at the time of advanced heart failure therapy, such as heart transplantation or left ventricular assist device (LVAD) therapy, by tissue histology [10]. In fact, diagnosis is especially challenging in CS cases with solitary cardiac involvement, often requiring several biopsies, targeted by cardiac imaging, for diagnosis [11]. Due to these barriers, new imaging modalities, such as cardiac magnetic resonance imaging (CMR) and cardiac positron emission tomography using ^18^F-Fluorodeoxyglucose (PET-FDG), are currently being increasingly utilized as alternative methods for the diagnosis of CS.

Characteristic findings of CS on CMR include the detection of nonviable tissue through patchy and multifocal late gadolinium enhancement (LGE), with sparing of the endocardial border in the basal segments, particularly of the septum and lateral wall [12]. In the context of sarcoidosis, the CMR myocardial T2 mapping signal is presumed to reflect active granulomatous inflammation, which is potentially reversible with appropriate treatment [13,14]. Notably, current studies support the inclusion of both LGE and abnormal T2 signals as complementary for the detection of active CS [13]. In the case of PET-FDG, focal or focal-on-diffuse FDG uptake patterns suggest active inflammation in CS [15,16]. Importantly, patients with either CMR or PET-FDG findings consistent with CS have a 4–19-fold higher risk of ventricular arrhythmias or all-cause mortality over 1.5–3 years of follow-up, with the highest risk in patients with a preserved ejection fraction (EF) [17].

Because diagnosis remains a challenge and no consensus exists on the detection, monitoring, and treatment of CS, the literature on CS mostly consists of single case reports or small case series [18,19,20,21]. The diagnosis and management of CS ultimately depend on expert consensus, with no recommendations validated by prospective data or clinical trials [22,23]. Indeed, three sets of expert consensus criteria are utilized currently for the diagnosis of CS. These include the World Association of Sarcoidosis and other Granulomatous Disorders (WASOG) organ assessment tool, published in 2014 and updated in 2020 by the American Thoracic Society Guidelines for Sarcoidosis Diagnosis [24,25]; the Japanese Ministry of Health & Welfare (JMHW) 2006 criteria revised by the Japanese Circulation Society (JCS) 2016 criteria [26]; and the 2014 Heart Rhythm Society (HRS) criteria [5,27], which are thought to have greater sensitivity, as they include both cardiac PET-FDG and CMR as acceptable imaging modalities, as well as responsiveness to glucocorticosteroid (GC) treatment [5,19,24,25,27,28,29,30].

In our study, we present a 5-year retrospective case series of CS patients, diagnosed based on the 2014 HRS criteria and followed by two tertiary referral medical centers. Our aim in presenting this case series is to describe the clinical and laboratory manifestations of CS, focusing on the utility of current imaging modalities, specifically CMR and cardiac PET-FDG, in CS diagnosis based on standardized diagnostic criteria for CS.

## 3. Methods

### 3.1. Study Population

This real-life retrospective case series was conducted in two tertiary referral medical centers (Carmel Medical Center (CMC) and Hadassah Medical Center (HMC)) and included patients diagnosed with systemic sarcoidosis with cardiac involvement from 1 November 2017 to 1 November 2022, based on the 2014 HRS Expert Consensus Recommendations on Criteria for the Diagnosis of CS [5,27]. In the 2014 HRS criteria, a ‘definite diagnosis of CS’ can be made based on histology from myocardial tissue, with the presence of non-caseating granulomas (NCGs) and no identifiable alternative cause for cardiac involvement. Alternatively, ‘probable CS’ (defined as >50% likelihood of CS) can be determined in the setting of a histological diagnosis of extra-cardiac sarcoidosis and the presence of one of several additional criteria, including GC- and immunosuppressant-responsive cardiomyopathy or heart block, unexplained left ventricular ejection fraction (LVEF) <40%, unexplained sustained VT, Mobitz type II, second- or third-degree heart block, patchy uptake on dedicated cardiac PET-FDG in a pattern typical of CS, LGE on CMR in a pattern consistent with CS, and/or positive gallium uptake in a pattern consistent with CS, following the exclusion of other causes for cardiac disease.

In our study, the definitions of ‘definite CS’ and ‘probable CS’ noted above were based on diagnostic data from hospital admissions and rheumatology and cardiology clinic visits in the two respective medical centers. All data were reviewed by TG and FK, who ascertained the diagnoses of ‘definite CS’ and ‘probable CS’ based on the 2014 HRS criteria. In cases of isolated cardiac involvement, where EMB was unavailable or non-diagnostic, the reviewers TG and FK considered a new diagnostic category that was also recently utilized by Rosenbaum et al. [31] of ‘presumed CS’, in which typical cardiac imaging findings consistent with CS based on the 2014 HRS criteria, along with at least one typical cardiac clinical presentation considered representative of CS in the 2014 HRS criteria, were both present.

### 3.2. Data Collection

Patient data, including demographic, clinical, laboratory, and imaging characteristics, were collected retrospectively from patient medical records, consisting of hospital admission records and rheumatology and cardiology clinic visit notes, by the study investigators (TG, FK, RA, JF, AH, NH, AK).

Demographic parameters were recorded regarding each patient, including age and sex; laboratory data, including biomarkers such as the C-reactive protein (CRP) level, troponin level, B-type natriuretic peptide (BNP) level, and angiotensin converting enzyme (ACE) level; pathology reports; radiology studies, including computed tomography (CT) scans, CMR, and cardiac PET-FDG; and data on cardiac devices (implantable cardioverter-defibrillator (ICD) and pacemaker (PM) implantation) and medical therapy, with an emphasis on GC and immunosuppressive medications.

Cardiac studies, including imaging and EMB results, were reviewed by the cardiologists AF and RA, as well as the radiologist MM and the pathologist ES, in the respective medical centers.

### 3.3. Ethical Considerations

This study was performed in accordance with the principles of the Declaration of Helsinki and was approved by the research ethics committees (institutional review boards) of the two participating medical centers (CMC-0125-22 at Carmel Medical Center, and HMO-0357-22 at Hadassah Medical Center).

## 4. Results

### 4.1. Study Participants

Our case series included 19 patients, 53% (*n* = 10) of whom were men, with a mean age (at the time of cardiac involvement) of 52.9 ± 11.8 (Supplementary Materials: Tables S1 and S2). In 63% (*n* = 12) of the cases, CS was the initial presentation of sarcoidosis, and in 63% (*n* = 12), high-grade (Mobitz type II second- or third-degree) AVB was the main cardiac manifestation of CS, although, in 42% (*n* = 8) of CS cases, tachyarrhythmias in the form of atrial fibrillation (AF) (*n* = 3) or VT (*n* = 5) occurred, with five cases presenting with concurrent tachyarrhythmia and an advanced degree of cardiac conduction delay. Notably, of the 12 patients with AVB, one patient presented with acute myocardial infarction and later with clinical myocarditis, and another patient with pericarditis. Overall, 42% (*n*= 8) of the patients presented with acute-onset heart failure, with three of these cases attributed to clinical myocarditis.

### 4.2. Patient Diagnosis

**Figure 1:**
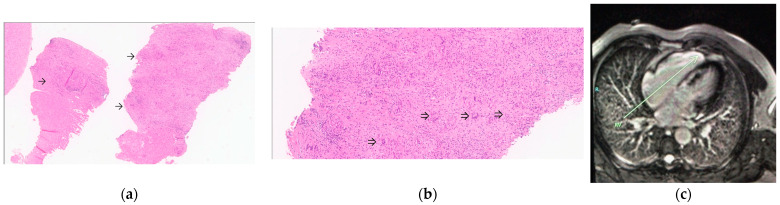

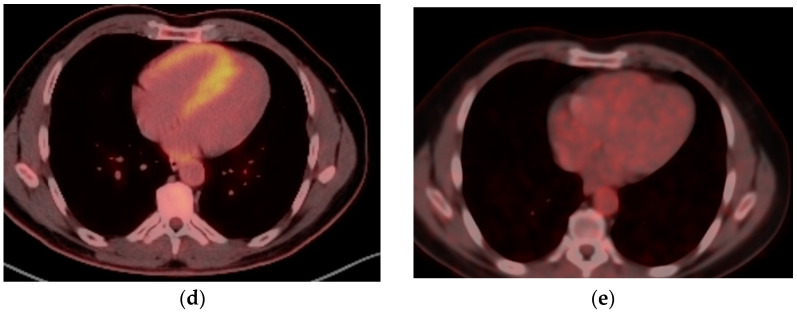
Evidence of sarcoidosis with cardiac involvement in patient M.Z. (case #14). (**a**) EMB showing NCGs (arrows) (H&E, magnification ×30). (**b**) High-power view of myocardial sections showing mononuclear inflammatory cells admixed with multinucleated giant cells (arrowheads) and interstitial fibrosis (H&E, magnification ×100). (**c**) CMR showing LGE in the interventricular septum and RV wall (long arrow). (**d**) Cardiac PET-FDG showing increased uptake in the LV infero-apical wall and in the septum. (**e**) Repeat cardiac PET-FDG 6 months after initiation of immunosuppression showing no active signal.

In our series, CMR and/or cardiac PET-FDG was obtained in 18/19 cases of systemic sarcoidosis with cardiac involvement. Diagnosis based on imaging—either CMR and/or cardiac PET-FDG—was possible in all 18 patients (one of whom also had supporting EMB) along with appropriate clinical criteria. In 13 of these cases, the diagnosis was determined as ‘probable CS’ based on an extra-cardiac biopsy consistent with sarcoidosis and at least one additional 2014 HRS criterion for CS. One additional case was designated as ‘probable CS’ based on characteristic histological findings in the extra-cardiac biopsy and high-degree AVB in a patient who refused cardiac imaging. In 16% (*n* = 3) of cases, cardiac involvement was solitary, and where EMB was unavailable or non-diagnostic, these cases were defined as ‘presumed CS’ based on the presence of a typical clinical presentation and cardiac imaging findings consistent with CS based on the 2014 HRS criteria. ‘Definite CS’ was diagnosed only in two cases based on EMB showing NCGs (Figure 1a–e).

Of all CS patients in this case series who underwent CMR + myocardial T2 mapping ± cardiac PET-FDG, 58% (*n* = 11) had active cardiac disease documented via T2 mapping on CMR and/or increased FDG absorption suggesting active inflammation by PET-FDG, while 32% (*n* = 6) had no evidence of active disease. Of this latter group of patients, all but one presented with high-grade AVB, whereas the remaining case presented with VT and heart failure.

When tested, the ACE level was elevated only in 14% (*n* = 2/14) and CRP in 50% (*n* = 9/18) of cases; troponin was elevated in 47% (*n* = 7/15) and BNP levels in 4/5 cases.

**Figure 2:**
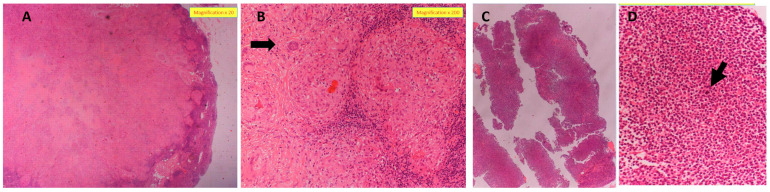
Patient S.Y. (case #10) with concurrent sarcoidosis and lymphoma (sarcoidosis–lymphoma syndrome). (**A**,**B**) Cervical lymph node biopsy supportive of sarcoidosis. (**A**) Cervical lymph node biopsy showing lymph node subtotally replaced by confluent non-necrotizing epitheloid granulomas, sarcoid-like, partially hyalinized (H&E, magnification ×20). (**B**) High magnification showing non-necrotizing sarcoid-like epitheloid granulomas with a few multinucleated giant cells (arrow) (H&E, magnification ×200). (**C**,**D**) Abdominal cavity (tru-cut biopsy) findings supportive of lymphoma in patient S.Y. (**C**) Cores of lymph tissue showing proliferation of small monomorphic B lymphocytes admixed with large activated lymphocytes. Immunohistochemically, these cells were positive for CD20 and BCL-2 and negative for other markers. Ki-67 proliferation index was approximately 15–20%. Findings consistent with low grade B-cell lymphoproliferative neoplasm, most probably, marginal-zone lymphoma (H&E, magnification ×20). (**D**) Cores of lymph tissue showing proliferation of small monomorphic B lymphocytes admixed with large activated lymphocytes (arrow) (H&E, magnification ×400). Of note, diagnosis of abdominal cavity lymphoma was also supported by bone marrow biopsy with 20% bone marrow involvement.

**Figure 3:**
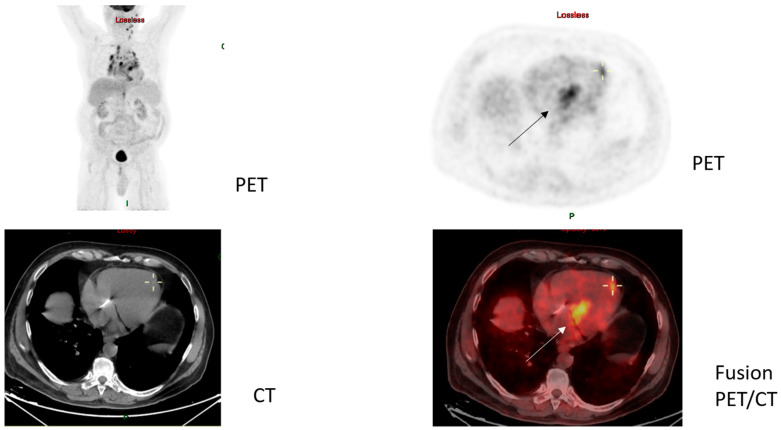
Total body PET-FDG images showing pathologic uptake in septum and cardiac apex, also seen on cardiac PET-FDG in patient S.Y. (case #10).

Importantly, our case series highlights the significance of combining data from both CMR and cardiac PET-FDG imaging studies to determine the disease activity of CS. This is noted, for instance, in case #10 of a 74-year-old male who presented to our clinic with an eyelid biopsy showing NCGs consistent with sarcoidosis (Figure 2A,B). Initial workup in his case only showed stage 1 pulmonary involvement and was not suggestive of cardiac disease; he was treated with a short prednisone taper and methotrexate (MTX). A year later, he was hospitalized for syncope due to a tri-fascicular block. A cardiac PM was inserted, and CMR, the only cardiac imaging modality available to us at that time, was completed, revealing no signs of cardiac involvement. Due to continued exertional dyspnea, he underwent further workup, which subsequently showed both the co-existence of marginal-zone lymphoma (a condition characterized in the medical literature as sarcoidosis–lymphoma syndrome [32,33], (Figure 2C,D)) and interventricular septal widening concerning for CS on TTE, with PET-FDG (Figure 3) showing typical findings of CS with uptake in the basal cardiac septum and apex of the left ventricle, necessitating the initiation of a high-dose GC taper starting at 40 mg/day and mycophenolate mofetil (MMF). During the cardiac follow-up, the patient also developed AF, the most common supraventricular arrhythmia seen in CS [34]. As noted in this case, active CS was not demonstrated and long-term immunosuppression for CS was not started until active cardiac involvement was evident by cardiac PET-FDG imaging, while not initially detected by previous CMR obtained at the time of development of the tri-fascicular block. Given the complexity of his comorbidities and full dependency on his PM, he has yet to undergo PM replacement to ICD.

In our case series, medical treatment in 17 out of 19 CS patients consisted of GC, and 79% (*n* = 15) were also prescribed MTX as a GC-sparing agent (Supplementary Materials: Tables S1 and S2a). Two individuals were not prescribed immunosuppressive agents because, in one case, sarcoidosis was not diagnosed until 6 years following the initial presentation with Mobitz II, and this individual has been asymptomatic since obtaining cardiac PM and is not interested in any further treatment. In another case, cardiac imaging revealed old fibrotic scarring without active granulomatous inflammation.

Agents used following MTX failure or intolerance in our case series included azathioprine (AZA), MMF, anti-tumor necrosis factor alpha (anti-TNFα) agents, or the anti-CD20 agent rituximab (RTX), with the latter given for a concurrent diagnosis of marginal cell lymphoma in case #10, as noted above. In 53% (*n* = 10) of the cases, an ICD was inserted when warranted, based on the HRS Expert Consensus Statement on the Diagnosis and Management of Arrhythmias Associated with CS [27], with an additional four cases involving initial PM placement when the diagnosis of sarcoidosis was not initially recognized. Subsequently, the PM was replaced by an ICD in one case, with the decision not to switch the other PMs to ICDs due to a lack of arrhythmias on cardiac monitoring following PM placement and a lack of active cardiac disease on cardiac imaging. Of note, to date, all patients in our case series remain alive, with no cases of mortality of any cause.

## 5. Discussion

In our retrospective, real-life case series of CS patients, conducted in two tertiary referral medical centers, we found that in the majority of cases, cardiac involvement was the initial, and occasionally the sole, manifestation involved in systemic sarcoidosis. Importantly, physicians relied heavily on modern imaging studies both for the diagnosis and management of these patients. Most cases of CS involved advanced AV conduction delays, with some cases also concurrently involving supraventricular arrhythmias, or, more commonly, VT.

Our results are in line with other case reports and case series of CS, in which most CS cases present with AV conduction abnormalities, with a noted prevalence of up to 62% in the literature [35], and with many cases occurring as initial presenting events in systemic sarcoidosis [18,19,20,21,36]. Furthermore, in accordance with the literature, we found fewer cases of tachyarrhythmias and heart failure and only one case of symptomatic pericardial involvement, which has rarely been reported in the literature [18,19,20,21].

Interestingly, unlike a previous report by Banba et al., which associated the presence of the uptake of gallium-67 citrate (Ga) in scintigraphy [36] as representative of active CS with AV conduction delays, in our study, AVB was not associated with active disease using T2 mapping on CMR and/or cardiac PET-FDG. Moreover, while Banba et al. noted that VT is a manifestation of late and more advanced cardiac involvement, we did find cases in which VT was associated with active CS based on cardiac imaging.

As in our case series, most CS cases in the literature were treated with a GC taper, with MTX used as the chief GC-sparing agent [18,19,20,21]. Prednisone has been shown retrospectively to reduce conduction abnormalities, although it was not shown in a study by Banba et al. to be effective in reducing ventricular tachyarrhythmias [36]. Importantly, GC treatment has also been shown to improve cardiac function and increase survival [37,38]. As the chief GC-sparing agent, MTX has been noted to be effective as a maintenance therapy in CS [39] and is currently being investigated in the two-arm clinical trial entitled The Cardiac Sarcoidosis Multi-Center Randomized Controlled Trial (CHASM CS-RCT; NCT03593759), consisting of GC + MTX vs. GC alone [40]. Other agents used in the case of GC failure or the development of adverse side effects due to MTX include AZA [41], leflunomide (LEF) [28], MMF [42,43], and cyclophosphamide (CYC) [44], as well as the newer TNF-alpha inhibitors, especially infliximab and adalimumab, which have recently been shown to be effective as GC-sparing therapies in refractory cases of CS [39,45]. According to our review of the literature, no clinical trial results currently exist for CS management, although a handful of clinical trials are currently recruiting patients for treatment strategies, including IL-1 blockade and the anti-granulocyte macrophage-colony stimulating factor (GM-CSF) agent namilumab (www.clinicaltrials.gov under ‘cardiac sarcoidosis’ accessed on 4 August 2022 and 14 April 2023).

Notably, not until this year have there been large registry-type studies published on CS and exploring the use of formal guideline criteria for CS diagnosis. For instance, in the recently published Japanese multicenter ILLUMINATE-CS registry data regarding 512 patients with CS [46], histological confirmation of CS (‘biopsy-proven CS’ or ‘definite CS’ according to the 2014 HRS criteria) was seen in 314/512 (61.3%) patients, while those with strongly suggestive clinical findings of CS without histological evidence of CS (classified as ‘clinical CS’ based on the 2016 JCS criteria or ‘probable CS’ according to the 2014 HRS criteria) constituted 198/512 (38.7%) of patients. The authors reporting data from this registry found that ‘clinical CS’ was associated with a higher prevalence of LV dysfunction, septal thinning, and positive PET-FDG uptake than ‘biopsy-proven CS’, leading the authors to conclude that ‘considering the worse clinical outcomes irrespective of the histological evidence, the diagnosis of clinical CS is justifiable if imaging findings suggestive of CS are observed.’ Similar findings were seen in a recent series of 383 sarcoidosis patients followed at the Mayo Clinic [31], with a lower percentage of patients (more comparable to that observed in our case series) of 59/383 (15.4%) classified as ‘definite CS’, and 223/383 (58.2%) with ‘probable CS’ (both diagnostic categories adjudicated based on the 2014 HRS criteria). Similar to our study, these authors added another diagnostic category in 101/383 (26.4%) cases with a clinical diagnosis of ‘presumed CS’ (of which 62 cases were of isolated CS and 39 of systemic CS) not determined based on formal guideline criteria, but instead relying on unexplained high-grade AVB or ventricular arrhythmia and findings suggestive of CS on either CMR or PET-FDG without any histologic evidence of sarcoidosis. These authors found the patients in the non-guideline criteria group of ‘presumed CS’ to have a poor prognosis comparable to those CS patients with histologic evidence of disease. As can be seen even in these two large, multicenter, registry-type studies, the diagnosis of CS in real-life practice relies heavily on imaging data.

Along these lines, CMR and PET-FDG were both used as complementary imaging modalities in our case series, not only for CS diagnosis but also to determine the medical and device therapeutic management of patients with CS, with the added advantage of the utilization of both imaging modalities over a single modality especially in determining disease activity. As reported in the literature, most of our CS cases were treated with GC tapers, with MTX used as the chief GC-sparing agent [18,19,20,21]. The need for and duration of immunosuppressive therapy in our CS cases were determined by cardiac imaging.

Limitations of our study: The aim of this retrospective study was to specifically examine a series of patients who were diagnosed with CS in our patient population over the past five years and to determine whether the novel imaging modalities utilized in the HRS criteria aid in the diagnosis of CS in real life. Thus, we did not examine the myriad of cases of extra-cardiac sarcoidosis that we evaluated over the past five years. Given the retrospective study design focusing on patients with clinical cardiac involvement, it is likely that other sarcoidosis patients with subclinical cardiac involvement were missed.

Although our case series was limited to 19 patients diagnosed with CS over the past 5 years only, we performed a detailed chart review of the medical data and relied on a specific set of diagnostic criteria to demonstrate the real-life utility of these criteria in CS diagnosis and management.

## 6. Conclusions

In summary, in our real-life case series utilizing the 2014 HRS diagnostic criteria, we found that cardiac involvement was the initial presentation of sarcoidosis in most cases, with physicians relying heavily on CMR and PET-FDG for the diagnosis and management of CS. The utilization of both imaging modalities was helpful in recognizing cardiac involvement and in evaluating whether cardiac involvement was active or consisted of granulomatous scar tissue, thus helping to determine whether immunosuppression was warranted. The use of the updated HRS criteria for CS diagnosis is important, especially given their emphasis on novel imaging modalities for the prompt recognition and management of this potentially life-threatening condition.

## Data Availability

The data used and/or analyzed during the present study are available from the corresponding author on reasonable request.

## Supplementary Materials

The following supporting information can be downloaded at: https://www.mdpi.com/article/10.3390/jcm12165278/s1, Table S1: Clinical cases of cardiac sarcoidosis: presentation, diagnosis, and treatment; Table S2: Clinical characteristics of cases of cardiac sarcoidosis.
