# Supplementary material for: Real-Life Utilization of Criteria Guidelines for Diagnosis of Cardiac Sarcoidosis (CS)
Source: J Clin Med. 2023 Aug 14;12(16):5278. doi: 10.3390/jcm12165278 (PMC10455608; doi:10.3390/jcm12165278)
Supplement: Supplementary file 1 [file jcm-12-05278-s001.zip › jcm-2478804-supplementary.pdf]

Table S1: Clinical cases of cardiac sarcoidosis: presentation, diagnosis, and treatment

Patient<br>Age<br>Sex	Clinical symptoms of cardiac involvement and initial cardiac workup	Diagnosis of cardiac sarcoidosis	Cardiac involvement following initial dx of sarcoidosis	Treatment	Extra-cardiac involvement
Case 1<br>**Probable CS**<br>71<br>F	Pericarditis<br>LAHB+CRBBB on ECG	- CMR with focal LGE in basal-antero-septal wall with less than 1% myocardial involvement and circular LGE of pericardium.<br>- Cardiac PET-FDG without cardiac uptake.<br>- -Normal ACE level, elevated CRP level.	2 years	- Colchicine, high dose ASA and<br>- Prednisone taper from 30 mg/day	**RETICULOENDOTHELIAL:** hilar LAD with uptake on PET-FDG with NCSs on EBUS-guided biopsy<br>**LUNG**: Pulmonary nodules, bronchiectasis<br>**MSK**: Arthritis, diffuse skeletal uptake on PET-FDG
Case 2<br>**Presumed CS**<br>54<br>F	Syncope and DOE<br>CAVB on ECG + WCT on monitor	- CMR with LGE in mid-wall basal septum, less than 1% uptake<br>- Only T1 signal seen without T2 signal, suggesting old granulomatous inflammatory damage<br>- Total body PET-FDG without cardiac uptake<br>- Normal ACE level, normal CRP level.	Initial presentation	-PM insertion	**ISOLATED CARDIAC**<br>Dx following COVID-19
Case 3<br>**Probable CS**<br>41<br>M	-DOE with bradycardia due to CAVB on ECG a month following NSTEMI and cCTA + stent<br>-2 years later, DOE with troponin elevation c/w myocarditis with normal cCTA	- CMR with mild basal septal thickening, focal transmural LGE in basal apical segment with mild decrease in global systolic function in RV<br>- Cardiac PM interrogation showing episodes of asymptomatic asystole 2 years later with TTE with EF=25% with global dyskinesia<br>- Normal ACE level, elevated troponin, elevated CRP level.	Initial presentation	- Initial PM insertion<br>- Prednisone taper from 60 mg/day and MTX 20 mg/week with subsequent d/c by patient and loss to follow-up<br>- 2 years later: PM replaced by ICD due to suspected CS<br>- Pulse IV steroids + prednisone taper from 60 mg/day + MTX 15 mg/week which was switched to AZA 150 mg/day due to side effects	**RETICULOENDOTHELIAL**: Mediastinal LAD on CT with NCGs on EBUS-guided biopsy<br>**NEUROLOGIC**: Peripheral nerve B/L drop foot
Case 4<br>**Probable CS**<br>62<br>F	Generalized weakness due to bradycardia<br>CLBBB +2:1 AVB on ECG + PMVT on monitor	- TTE with diastolic dysfunction + asynchrony of septum c/w CLBBB<br>- CMR with increased signal on T1 mapping (fibrosis) in apical septum c/w fibrosis, unable to complete Gadolinium injection to check for LGE due to SOB.	12 years	- Short prednisone taper<br>- ICD insertion when presenting with suspected cardiac involvement	**RETICULOENDOTHELIAL**: Generalized LAD on CT with NCGs on EBUS-guided biopsy, splenomegaly<br>**LUNG**: Pulmonary nodules with uptake on PET-FDG, bronchiectasis<br>**LIVER**: NCGs on liver biopsy

		- Cardiac PET-FDG without myocardial uptake.<br>- Normal ACE level around diagnosis of CS, was chronically elevated up to 95 prior to CS, normal CRP level.			**HEMATOLOGIC**: bone marrow biopsy showing NCGs.
Case 5<br>**Presumed CS**<br>47<br>F	DOE and weakness due to bradycardia<br>CAVB on ECG	- CMR with mild LGE in infero-septal (basal) wall involving 1% of myocardium with right atrial enlargement.<br>- Cardiac PET-FDG with poor preparation-non indicative of activity.<br>- Normal ACE level, normal CRP level	Initial presentation	- Prednisone taper from 60 mg/day+ MTX 10 mg/week<br>- ICD insertion<br>- Loss to follow-up.	**ISOLATED CARDIAC**
Case 6<br>**Probable CS**<br>61<br>F	DOE and weakness 2/2 bradycardia<br>Small pericardial effusion<br>CAVB on ECG<br>Normal coronary CT	- CMR with LGE in basal septum, no T1 or T2 signal, suggesting old granulomatous inflammatory damage<br>- Total body PET-FDG with no cardiac uptake.<br>- Normal ACE level, elevated CRP level.	Initial presentation	- Prednisone taper from 40 mg/day + MTX<br>- ICD insertion<br>- Loss to follow-up.	**RETICULOENDOTHELIAL**: Mediastinal LAD with abnormal uptake on PET-FDG with NCGs on EBUS-guided biopsy<br>**LUNG:** pleural effusion and GGO in lower lobes<br>**LIVER**: focal liver uptake on PET-FDG
Case 7<br>**Presumed CS**<br>56<br>M	DOE, weakness and syncope<br>Alternating CRBBB, CLBBB and PR prolongation on monitor, CAVB on ECG.<br>Mild troponin elevation with normal coronary CT.	- CMR with hypokinesis of apex and lateral wall, septal thickening. Positive T1 signal (interstitial fibrosis) and T2 signal (myocardial edema) with epicardial LGE in antero-septal wall and in lateral wall with 8% myocardial involvement.<br>- Cardiac PET-FDG with elevated physiologic uptake in myocardium without ability to discern pathologic uptake<br>- Normal ACE level, mild troponin elevation, normal CRP level.	Initial presentation	- Prednisone taper from 40 mg/day + plan to add MTX by provider<br>- ICD insertion	**ISOLATED CARDIAC**
Case 8<br>**Probable CS**<br>40<br>F	Intermittent CP with DOE, palpitations.<br>Normal TTE.	- CMR with LGE in mid-wall of basal septum with 1% myocardial involvement.<br>- Cardiac PET-FDG with perihilar LAD without uptake, cardiac apical uptake, no septal uptake.<br>- Normal ACE level, elevated CRP level.	Initial presentation	- Prednisone taper from 40 mg/day + HCQ for joints<br>- Switched to MTX+SSZ with continuation of low dose prednisone for joints	**RETICULOENDOTHELIAL:** Mediastinal LAD on CT with NCGs on EBUS-guided biopsy<br>**LUNG:** Pulmonary nodules<br>**MSK**: Arthritis<br>-Crohn's disease dx a few months following sarcoidosis
Case 9<br>**Probable CS**<br>56<br>M	Intermittent CP with SOB, palpitations, signs and symptoms of heart failure with pulmonary edema.<br>TTE with narrow complex	- CMR with severely reduced LVEF with mild-mod reduction in RV function, LGE in mid-wall of basal septum, c/w dilated non-ischemic CMP.<br>- Cardiac PET-FDG without uptake<br>- Previous CMR (2 years prior) with mild lateral wall hypokinesis, LGE in lateral	2-4 years following initial diagnosis (dx 4 years following initial dx but suspicion arose 2 years prior)	- Prednisone taper from 15 mg/day + MTX 20 mg/week on dx, followed by Depo Medrol pulse IV 1 g X 3 days and AZA for a short time replaced by MMF up to 2 g/day (dose	**RETICULOENDOTHELIAL:** Perihilar and mediastinal LAD on CT with NCGs on EBUS-guided biopsy<br>**LUNG**: Interstitial changes and GGO<br>**MSK**: Muscle biopsy with NCGs

	tachycardia, EF=25%, LVEDD 61 mm (enlarged) with mild-mod MR (prior TTE with normal LVEF=60%). Repeat ECG with NSR with PRWP and with LAHB.	wall with 6% cardiac myocardial involvement- attributed at the time to AMI though cCTA with no significant cardiac lesions.<br>- Normal ACE level around CS dx, elevated only once to 148 prior (normal <70), Troponin elevation to 300, pro-BNP elevation>3000, elevated CRP level.		lowered to 1.5 g/day d/t hypogammaglobulinemia)<br>- ICD inserted due to heart failure and NSVT, SVT noted on ICD interrogation	
Case 10<br>**Probable CS**<br>69<br>M	Hospitalization for syncope with asystole for 4-5 sec. Follow-up ECG with tri-fascicular block. AF developed later during follow-up.	- TTE with widened IVS 1.3 (normal until 1.1).<br>- CMR with suspected LGE in focal infero-septal-mid basal septum with septal thickening.<br>- Cardiac PET-FDG with pathologic cardiac septal and apical uptake.<br>- Repeat PET-FDG 6 months later without cardiac uptake after initiation of immunosuppression, perihilar and mediastinal lymph node uptake still present.<br>- Normal ACE level, normal CRP level.	1.5 years following dx	- Prednisone taper on dx followed by MTX up to 20 mg/week<br>- PM insertion<br>- Chemotherapy for NHL (included RTX and Depo Medrol) following dx of sarcoidosis.<br>- Prednisone later restarted with MMF 2 g/day following cardiac PET-FDG with dx of CS.<br>- Underwent ablation for AF	**RETICULOENDOTHELIAL**: PET-FDG with uptake in submandibular, cervical and mediastinal lymph nodes, cervical LN biopsy with sarcoid-like NCGs<br>**EYE**: focal uptake in L eye orbit on PET-FDG<br>**LIVER**: focal liver uptake on PET-FDG<br>**SKIN**: L lower eyelid biopsy with NCGs
Case 11<br>**Probable CS**<br>50<br>M	Worsening DOE and CP with neg cardiac cath, orthopnea	- TTE with normal EF<br>- CMR with low-normal cardiac function, increased T1 signal in medial septum and focal LGE in medial septum.<br>- Cardiac PET-FDG with no cardiac uptake.<br>- Normal ACE level, normal Pro-BNP level.	1.5 years following dx	Prednisone taper from 30 mg/day with MTX 15 mg/week<br>-Switched to AZA 100 mg/day for several months due to lack of efficacy, d/c due to transaminitis<br>-Switched to infliximab biosimilar and later to adalimumab biosimilar due to pruritus on infliximab biosimilar	**RETICULOENDOTHELIAL**: hilar LAD on CT with NCGs on EBUS-guided biopsy<br>**LUNG**: pulmonary nodules with uptake in reticulonodular infiltrates on PET-FDG
Case 12<br>**Definite CS**<br>42<br>M	-Hospitalized for VT with SCD<br>-Cardiac cath with normal coronaries<br>-TTE with severe LV dysfunction (EF=30-35%)	- EMB showing NCGs<br>- CMR: LV and RV LGE and increased T1 and T2 signals, compatible with myocarditis.<br>- Cardiac PET-FDG- diffuse myocardial uptake with resolution of inflammation on therapy<br>- Elevated ACE level 57 U/L (normal<52)<br>- Elevated troponin and pro-BNP levels, normal CRP level.	Initial presentation	-Prednisone taper from 60 mg/day+ MTX 15 mg/week<br>-ICD insertion	**RETICULOENDOTHELIAL**: LAD with abnormal uptake in supra- and infra-diaphragmatic lymph nodes on PET-FDG<br>Skin: Pruritus, responsive to therapy

Case 13<br>**Probable CS**<br>51<br>M	- Hospitalized for syncopal episode<br>- ECG: Mobitz II/cAVB<br>- EPS: Severe his-purkinje disease with intrahissian conduction block<br>- TTE: mild LVH, non-obstructive systolic anterior motion of mitral chordae<br>- AF appeared 8 months into Rx	- EMB showing a non-specific mononuclear infiltrate<br>- CMR: LGE in the interventricular septum<br>- Cardiac PET-FDG: heterogenous uptake in the LV, with dominant uptake in the septum, diffuse hypermetabolic LAD; repeat PET-FDG 5 months later showed resolution of inflammation, Rx discontinued 12 months after initiation of Rx with repeat 1-year cardiac PET-FDG again showing activity, including cardiac uptake<br>- Elevated ACE level 100 U/L (normal<52)<br>- Normal troponin level, elevated CRP level.	Initial presentation	- Prednisone taper from 60 mg/day + MTX up to 20 mg/week<br>- ICD insertion<br>- Underwent ablation for AF	**RETICULOENDOTHELIAL**: Mediastinal LAD on CT with NCGs on EBUS-guided biopsy<br>**MSK**: Arthralgia
Case 14<br>**Definite CS**<br>44<br>M	-Hospitalized for CP<br>-ECG: tri-fascicular block<br>-Holter: 2 events of MOBITZ 1<br>-TTE: asymmetric septal hypertrophy, mild RVH	- EMB showing NCGs<br>- CMR: LGE in the septum, RV, inferior and posterior walls of LV<br>- Cardiac PET-FDG: Increased uptake in the LV infero-apical wall and in the septum<br>- Repeat cardiac PET-FDG 6 months after initiation of immunosuppression showed no active signal.<br>- Normal troponin level, normal CRP level.	Initial presentation	-Prednisone taper from 60 mg/day + MTX 15 mg/week	**RETICULOENDOTHELIAL:** Supra- and infradiaphragmatic LAD with uptake on PET-FDG<br>**RENAL**: Hypercalciuria<br>**MSK**: Arthralgia
Case 15<br>**Probable CS**<br>67<br>F	-Hospitalized for DOE, peripheral edema<br>-TTE: new, severely reduced LV function with moderately reduced RV function and severe PHTN<br>-On monitor: many APBs	- CMR: LGE and increased T2 signal in LV<br>- Cardiac PET-FDG: No uptake.<br>- Normal troponin level, normal CRP level.	3 years	- Prednisone taper from 60 mg/day + MTX 15 mg/week<br>- ICD insertion	**RETICULOENDOTHELIAL:** LAD on CT<br>**LUNG:** interstitial changes on CT<br>**LIVER**: NCGs on liver biopsy<br>**RENAL**: Hypercalcemia<br>**SKIN**: non-specific skin rash responsive to therapy
Case 16<br>**Probable CS**<br>25<br>M	-Hospitalized for dyspnea<br>-ECG: normal<br>-TTE: segmental wall motion abnormalities, mainly infero-septal hypokinesis at the basal and mid LV with EF 50-55%	- CMR: LGE and increased T2 signal in LV; normal systolic function<br>- Cardiac PET-FDG: mediastinal and hilar LAD, lung infiltrates; no signs of cardiac disease; bilateral hypermetabolic pleural uptake<br>- Normal troponin, elevated CRP level.	Initial presentation	-Prednisone taper from 40 mg/day+ MTX 15 mg/week	**RETICULOENDOTHELIAL:** Perihilar and mediastinal LAD on CT with NCGS on EBUS-guided biopsy<br>**LUNG**: pulmonary infiltrates<br>**RENAL**: Hypercalciuria<br>**MSK**: Arthralgia, myalgia

Case 17<br>**Probable CS**<br>52<br>M	-Hospitalized for dyspnea with transudative pleural effusion<br>-TTE: Diastolic HF, EF 50-55%<br>-ECG: sinus tachycardia<br>-EPS-PVCs	- CMR: LGE and increased T2 signal in LV with normal systolic function; small pericardial effusion<br>- Cardiac PET-FDG :Non-specific septal and near-apical uptake; repeat PET-FDG 14 months following initiation of immunosuppression: resolution of inflammatory findings.<br>- Normal troponin, elevated CRP level.	A few months following uveitis	- Prednisone taper from 60 mg/day + MTX 15 mg/week<br>- Steroid eye gtt	**RETICULOENDOTHELIAL:** Increased uptake in hilar and mediastinal LAD on PET-FDG with NCGs on EBUS-guided biopsy<br>**LUNG**: parenchymal involvement, small pleural effusion<br>**EYE**: Left anterior uveitis<br>**MSK**: diffuse uptake in lower limb muscles and soft tissues on PET-FDG, a right iliac lytic bone lesion on PET-FDG, NCGS on thigh muscle biopsy<br>**SKIN**: skin nodules
Case 18<br>**Probable CS**<br>61<br>F	-Admission for syncope<br>-Mobitz type II	-Normal ACE, normal troponin level, normal CRP level.	Initial presentation, but was initially thought of as idiopathic cardiac conduction abnormality and only 6 years later was extra-cardiac involvement observed and diagnosis of sarcoidosis was made	- Cardiac PM	**RETICULOENDOTHELIAL:** Mediastinal and hilar LAD on CT with NCGs on EBUS-guided biopsy<br>**RENAL**: hypercalciuria with hypervitaminosis D
Case 19<br>**Probable CS**<br>57<br>F	-Dyspnea and palpitations, with sustained VT on ECG<br>-New-onset AF 9 months prior<br>-TTE- EF 40-44%, RV dilatation and dysfunction, LA dilatation, and mild-moderate MR	- CMR: LGE in the mid-myocardial and sub-epicardial septum and lower RV wall<br>- PET-FDG: pathologic and heterogenous myocardial uptake, including septal uptake.<br>- Repeat TTE within 14 months showed EF 45-50%<br>- Repeat PET-FDG after 14 months showed a decrease in myocardial uptake on therapy.<br>- Normal troponin and CPK levels, elevated CRP level.	Initial presentation	- Prednisone 50 mg/day, tapered slowly over months<br>- MTX 15 mg/week<br>- ICD insertion	**RETICULOENDOTHELIAL:** diffuse supra- and subdiaphragmatic LAD with abnormal uptake on PET-FDG with NCGs on EBUS-guided biopsy<br>**LUNG:** pulmonary infiltrates and interstitial changes<br>**RENAL**: Hypercalcemia and hypercalciuria<br>**MSK**: Arthralgia, jaw uptake on PET-FDG<br>**SKIN**: non-specific skin rash responsive to therapy

Abbreviations: ACE=angiotensin converting enzyme, AF=atrial fibrillation, AMI=acute myocardial infarction, APBs=atrial premature beats, AVB=atrioventricular block (CAVB=complete AVB) , ASA=aspirin, AVNRT=atrioventricular node reentrant tachycardia, AZA=azathioprine, B/L=bilateral, cCTA=cardiac CT angiography, CLBBB, complete left bundle branch block, CMP=cardiomyopathy, CMR=cardiac magnetic resonance imaging, CP=chest pain, CRBBB=complete right bundle branch block, CRP=C-reactive protein, CS=cardiac sarcoidosis, CT=computer tomography, c/w=consistent with, d/c=discontinued, DOE=dyspnea on exertion, dx=diagnosis, EBUS=endobronchial biopsy, ECG=electrocardiogram, EF=ejection fraction, EMB=endomyocardial biopsy, EPS=electrophysiological study, F=female, , GGO=ground glass opacities, GI=gastrointestinal, HCQ=hydroxychloroquine, HF=heart failure, ICD=implantable cardioverter-defibrillator, ILD=interstitial lung disease, L=left, LAD=lymphadenopathy, LAHB=left anterior hemiblock, LGE=late gadolinium enhancement, LN=lymph nodes, LV=left ventricle, LVEDD=left ventricle end diastolic volume, M=male, MMF=mycophenolate mofetil, MR=mitral regurgitation, MSK=musculoskeletal, MTX=methotrexate, NCGs=non-caseating granulomas, NHL-non-Hodgkin's lymphoma, NSR=normal sinus rhythm, NSTEMI=non ST elevation MI, NSVT=non-sustained VT, PET-FDG=positron emission tomography using 18F-Fluorodeoxyglucose, PHTN=pulmonary hypertension, PM=pacemaker, PMVT=polymorphic VT, pro-BNP=pro-bone natriuretic peptide, PRWP=poor R wave progression, PVCs=premature ventricular contractions, RTX=rituximab, RV=right ventricle, Rx=treatment, SCD=sudden cardiac death, SOB=shortness of breath, SSZ=sulfasalazine, SVT=supraventricular tachycardia, TTE=transthoracic echocardiogram, VT=ventricular tachycardia, WCT=wide complex tachycardia

Table S2: Clinical characteristics of cases of cardiac sarcoidosis																				
				Certainty of CS diagnosis based on criteria			CS disease activity			Cardiac clinical presentation							Clinical biomarker elevation			
**Patient**	**Age**	**Sex**	**Ethnicity**	**Definite CS**	**Probable CS**	**Presumed CS**	**Active disease**	**Inactive disease**	**Initial presentation**	**Heart block**	**AF**	**VT**	**AMI**	**Pericarditis**	**Myocarditis**	**HF**	**ACE**	**CRP**	**Troponin**	**BNP**
**Case 1**	71	F	J		1			1		1				1				1	1	N/A
**Case 2**	54	F	A			1		1	1	1		1								N/A
**Case 3**	41	M	J		1		1		1	1			1		1	1		1	1	1
**Case 4**	62	F	J		1			1		1		1								N/A
**Case 5**	47	F	A			1	N/A	N/A	1	1										N/A
**Case 6**	61	F	J		1			1	1	1								1	1	N/A
**Case 7**	56	M	A			1	1		1	1					1				1	N/A
**Case 8**	40	F	J		1		1		1									1	N/A	N/A

**Case 9**	56	M	J		1			1		1		1			1	1		1	1	1
**Case 10**	69	M	J		1		1			1	1								1	1
Case 11	50	M	A		1			1								1		N/A	N/A	
Case 12	42	M	J	1			1		1			1			1	1	1		1	1
Case 13	51	M	J		1		1		1	1	1						1	1		N/A
Case 14	44	M	A	1			1		1	1							N/A			N/A
Case 15	67	F	J		1		1									1	N/A			N/A
Case 16	25	M	A		1		1		1							1	N/A	1		N/A

Case 17	52	M	J		1		1									1	N/A	1	N/A	N/A
Case 18	61	F	J		1		N/A	N/A	1	1									N/A	N/A
Case 19	57	F	A		1		1		1		1	1				1	N/A	1		N/A
Total	Avg Age ±SD 52.9± 11.8	F=9 M=10	J=12 (63.2%) A=7	2	14	3	11/17	6/17	12	12	3	5	1	1	4	8	2/14	9/18	7/15	**4/5**

Abbreviations: A=Arab, ACE=angiotensin converting enzyme, AF=atrial fibrillation, AMI=acute myocardial infarction, Avg=average, BNP=B-type natriuretic peptide, CRP=C-reactive protein, CS=cardiac sarcoidosis, F=female, HF=heart failure, J=Jewish, M=male, N/A=Not available, SD=standard deviation, VT=ventricular tachycardia.

Table S2a: Clinical characteristics of cases of cardiac sarcoidosis-continued

	Certainty of CS diagnosis based on criteria			CS disease activity		Medications									Device implantation/ Arrhythmia management		
**Patient**	**Definite CS**	**Probable CS**	**Presumed CS**	**Active disease**	**Inactive disease**	**GC**	**Colchicine**	**HCQ**	**SSZ**	**MTX**	**AZA**	**MMF**	**ANTI-TNFα**	**RTX (ANTI-CD20)**	**ICD**	**PM**	**Ablation**
**Case 1**		1			1	1	1										
**Case 2**			1		1	Prescribed Not taken				Prescribed Not taken						1	
**Case 3**		1		1		1				1	1				1 (switch)	1	
**Case 4**		1			1	1									1		
**Case 5**			1	?	?	1				1					1		
**Case 6**		1			1	Prescribed Not taken				Prescribed Not taken					1		
**Case 7**			1	1		1				1					1		
**Case 8**		1		1		1		1	1	1							

**Case 9**		1			1	1						1			1		
**Case 10**		1		1		1				1		1		1		1	1
**Case 11**		1			1	1				1	1		1				
**Case 12**	1			1		1				1					1		
**Case 13**		1		1		1				1					1		1
**Case 14**	1			1		1				1					1		
**Case 15**		1		1		1				1							
**Case 16**		1		1		1				1							

**Case 17**		1		1		1				1							
**Case 18**		1		?	?												
**Case 19**		1		1		1				1					1	1	
**Total**	2	14	3	11/17	6/17	16/18	1	1	1	13/15	2	2	1	1	10	4	2

Abbreviations: AZA=azathioprine, CS=cardiac sarcoidosis, GC=glucocorticosteroids, HCQ=hydroxychloroquine, ICD=implantable cardioverter-defibrillator, MMF=mycophenolate mofetil, MTX=methotrexate, PM=pacemaker, RTX=rituximab, SSZ=sulfasalazine
